# Supplementary material for: Attentional reorientation along the meridians of the visual field: Are there different neural mechanisms at play?
Source: Hum Brain Mapp. 2020 Jun 11;41(13):3765–80. doi: 10.1002/hbm.25086 (PMC7416051; doi:10.1002/hbm.25086)
Supplement: Supplementary file 1 — Appendix S1 Supporting Information [file HBM-41-3765-s001.docx]

# MRI – Preprocessing

The following description of the preprocessing was automatically generated (see <http://fmriprep.readthedocs.io/en/1.1.1/workflows.html>) and minimally adapted.

The preprocessing of functional and anatomical data was performed using FMRIPREP version 1.1.1 (Esteban et al., 2018, 2019, RRID:SCR_016216), a Nipype (RRID:SCR_002502, Gorgolewski et al., 2011, 2017) based tool, run as a docker-image. Each T1-weighted volume (T1w) was corrected for intensity non-uniformity using N4BiasFieldCorrection v2.1.0 (Tustison et al. 2010) and skull-stripped using antsBrainExtraction.sh v2.1.0 (using the OASIS template). Spatial normalization to the ICBM 152 Nonlinear Asymmetrical template version 2009c (Fonov et al. 2009, RRID:SCR_008796) was performed through nonlinear registration with the antsRegistration tool of ANTs v2.1.0 (Avants et al. 2008, RRID:SCR_004757), using brain-extracted versions of both T1w volume and template. Brain tissue segmentation of cerebrospinal fluid (CSF), white-matter (WM), and gray-matter (GM) was performed on the brain-extracted T1w using fast (Zhang, Brady, and Smith 2001, FSL v5.0.9, RRID:SCR_002823).

Functional data were slice-time corrected using 3dTshift from AFNI v16.2.07 (Cox 1996, RRID:SCR_005927) and motion-corrected using mcflirt (FSL v5.0.9, Jenkinson et al. 2002). "Fieldmap-less" distortion correction was performed by co-registering the functional image to the same-subject T1w image with intensity inverted (Wang et al. 2017), constrained with an average fieldmap template (Treiber et al. 2016), implemented with antsRegistration (ANTs). This procedure was followed by co-registration to the corresponding T1w using boundary-based registration (Greve and Fischl 2009) with 9 degrees of freedom, using flirt (FSL). Motion correcting transformations, field distortion correcting warp, BOLD-to-T1w transformation, and T1w-to-template (MNI) warp were concatenated and applied in a single step using antsApplyTransforms (ANTs v2.1.0) using Lanczos interpolation.

Frame-wise displacement (Power et al. 2014) was calculated for each functional run using the implementation of Nipype.

Many internal operations of FMRIPREP use Nilearn (Abraham et al. 2014, RRID:SCR_001362), principally within the BOLD-processing workflow. For more details of the pipeline, see <http://fmriprep.readthedocs.io/en/1.1.1/workflows.html>.

# Eye Tracking Analysis

When performing the online recording of eye movements during the experiment, we suffered from several technical issues and difficulties, so that only a subset of the data could be analyzed.

## Methods

We used an EyeLink® 1000 (SR Research) system to record the eye movements of our participants while they performed the spatial cueing task in the MR-scanner. The infrared camera was placed behind the participants, and their eye-movements were monitored via an infrared capable mirror and recorded at a sampling rate of 500 Hz. Because of the difficult recording environment (for example, large shadows close to the participant’s eyes due to the head-coil and the use of lenses to correct for the participant’s vison), we could not follow a uniform protocol for data collection and calibration.

The collected raw eye-movement data was converted from the EyeLink® data format (.edf) to asci (.asc) files. During the conversion, raw eye-movements were transformed to gaze-coordinates in pixels. The python package cili (Acland and Wallis 2016) was used to extract and load the data for further processing in Python 3.7, utilizing the scipy-stack (Jones et al. 2001).

First, we epoched the eye-tracking data into single trials (250 ms before cue-onset until 400 ms after target onset). Each trial was further subdivided into three phases. The “pre-trial” phase, which lasted from 250 ms before cue onset until cue onset, served as baseline fixation period. The second (“cue”) phase comprised the time from cue onset until target onset (600 ms to 800 ms duration). The “target” phase was defined from target onset until 400 ms after.

The eye-data were cleaned by removing not-recorded values (i.e., NaN) and gaze coordinates exceeding the screen coordinates (0 > x > 1279, 0 > y > 799). If more than 10% of data for any of the three phases (assuming 600 ms duration for the “Cue” period) were removed, the whole trial was discarded.

We assumed that the participants were focusing on the screen’s center in the “Pre-trial” phase. Thus, we used data from this period to re-align the gaze coordinate. The latter was achieved by subtracting the difference between the screen’s center coordinates (x = 640, y = 400) and the robust mean of the pre-stimulus phase (mean of the data points between the 10^th^ and 90^th^ percentile).

Before statistical analyses, we discarded all datasets that had less than 100 valid or less than ten invalid trials. Furthermore, the data of a participant was only included if data of both sessions were present.

The final sample included eye-movement data of 17 out of the 27 participants.

Table 1: Number of trials retained in the eye-tracking analyses.

| Run | TrialType | Number of trials (included) | |
| --- | --- | --- | --- |
|  |  | M | SD |
| Horizontal | invalid | 37.24 | 2.68 |
|  | valid | 150.35 | 8.94 |
| Vertical | invalid | 35.29 | 2.97 |
|  | valid | 144.35 | 12.17 |

### Analyzing Saccades

We used two different estimates to analyze saccades. As a first estimate, we defined that the cue- or the target-phase contained a saccade towards the target position if eye movements along the x-axis for horizontal runs (y-axis for vertical runs) exceeded 50% of the distance between the central fixation cross and the middle of the left (lower) or right (upper) target box (“Eye-50”). Additionally, we also used the automatic saccade classification by the EyeLink® software (“EyeLink”). Saccades were determined using three different thresholds for motion (0.1 °), velocity (30 °/s), and acceleration (8000 °/s2), based on the cognitive preset described in the EyeLink® manual (http://sr-research.jp/support/EyeLink 1000 User Manual 1.5.0.pdf). Here we used the “SSACC” (start-saccade) and “ESACC” (end-saccade) messages in the EyeLink® as time-intervals. If any data point of the cue- or target-phase was contained in any of the saccade intervals, we assumed that a saccade was made in the corresponding phase.

We then calculated the proportion of trials containing a saccade for each participant, cueing-condition, and run. The proportion of trials containing saccades was then submitted to a 2 (cueing-condition) x 2 (run) Bayes Factor ANOVA (Morey and Rouder 2018) to test for possible differences between the factors of interest; participant was included as a random factor. We also calculated linear mixed effects models in R (R Core Team 2018) using lmertest (Kuznetsova, Brockhoff, and Christensen 2017) with participant as random intercept, using the same 2 x 2 design. Both analyses were done separately for the cue- and target-phase.

Pearson’s correlation coefficient between proportions of saccades between “EyeLink” and “Eye-50” in the cue-phase equaled 0.791 (p < 0.001), and 0.826 (p < 0.001) in the target-phase.

## Results

The results (see Table 3) provided no evidence for a significantly different distribution of saccades between the two runs, nor between the different cueing-conditions, nor their interactions. While none of the coefficients of the linear mixed effects model was statistically significant, the BF_ANOVAs often did not provide solid evidence against an effect. Still we are confident that participant’s eye-movements are not driving the effects (or the absence thereof) in the main analyses.

Table 2: Descriptive statistics (mean and standard deviation) for the proportion of trials containing a saccade.

| Phase | Run | Condition | Eye-50  M ± SD | EyeLinkSaccade  M ± SD |
| --- | --- | --- | --- | --- |
| Cue | Horizontal | invalid | 0.080 ± 0.197 | 0.310 ± 0.220 |
|  |  | valid | 0.077 ± 0.216 | 0.296 ± 0.219 |
|  | Vertical | invalid | 0.099 ± 0.206 | 0.322 ± 0.182 |
|  |  | valid | 0.095 ± 0.212 | 0.308 ± 0.219 |
| Stimulus | Horizontal | invalid | 0.098 ± 0.231 | 0.162 ± 0.210 |
|  |  | valid | 0.083 ± 0.228 | 0.101 ± 0.100 |
|  | Vertical | invalid | 0.110 ± 0.238 | 0.163 ± 0.202 |
|  |  | valid | 0.098 ± 0.228 | 0.121 ± 0.107 |

Table 3: Results of BF_ANOVAs and linear mixed effects models. The values in the first position are the BF_01_ in favor of the baseline model (based on the random intercept). The BF-ANOVA models all included the participant as random factor. P-values are the results for the different coefficients in the linear mixed effects model, where the p-values in the last column only describe the interaction of condition * run.

| Phase | Definition | Condition | Run | Condition + Run | Condition + Run + Condition * Run |
| --- | --- | --- | --- | --- | --- |
| Cue | Eye-50 | 3.77 (p = 0.792) | 3.25 (p = 0.135) | 2.13 | 0.44 (p = 0.975) |
|  | EyeLink | 0.61 (p = 0.626) | 3.41 (p = 0.642) | 2.16 | 3.72 (p = 0.988) |
| Stimulus | Eye-50 | 2.34 (p = 0.342) | 10.82 (p = 0.450) | 4.81 | 1.66 (p = 0.909) |
|  | EyeLink | 7.14 (p = 0.059) | 32.79 (p = 0.986) | 2.32 | 4.54 (p = 0.663) |


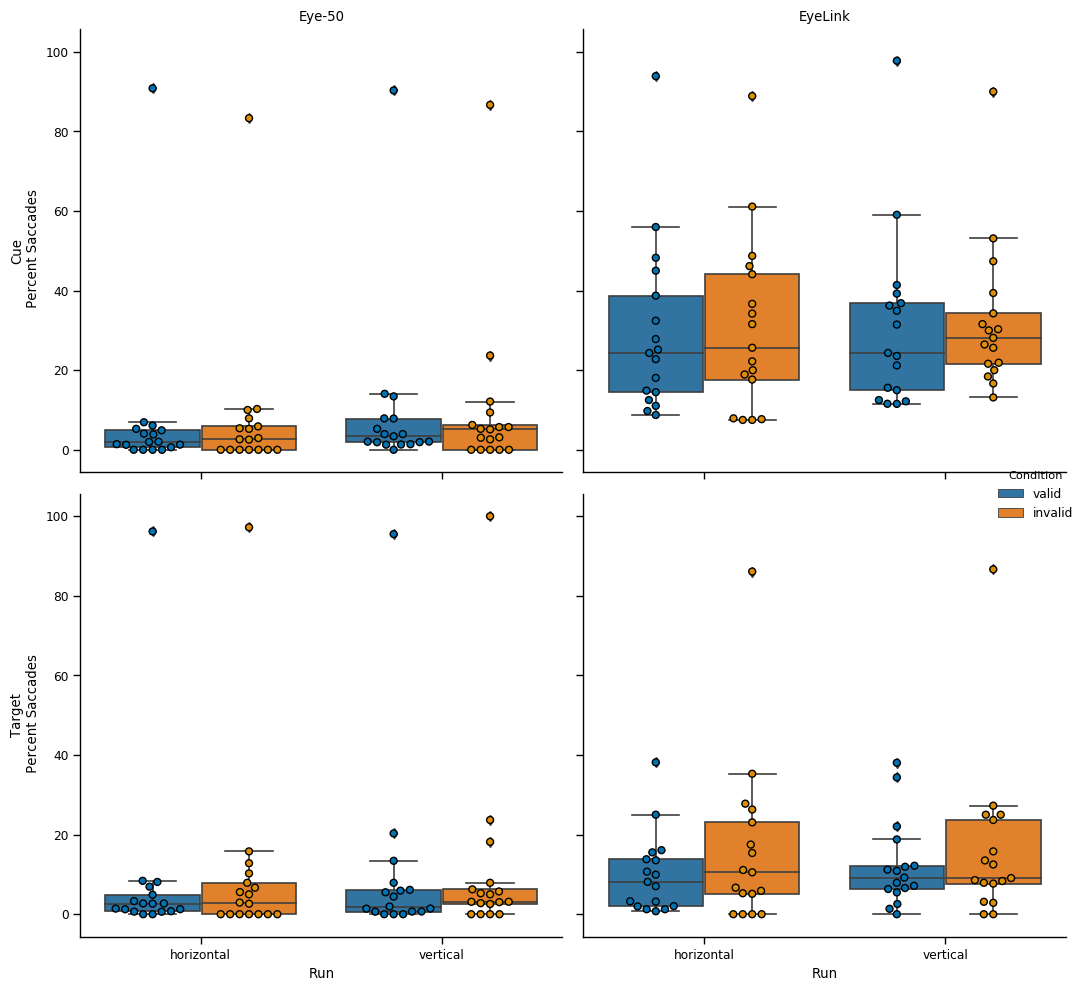


Figure 1: Visualization of the percentage of saccades during the experiment. Classical boxplots displaying the mean and the interquartile range (IQR) are used. Outlier were defined using the 1.5 * IQR criterion.


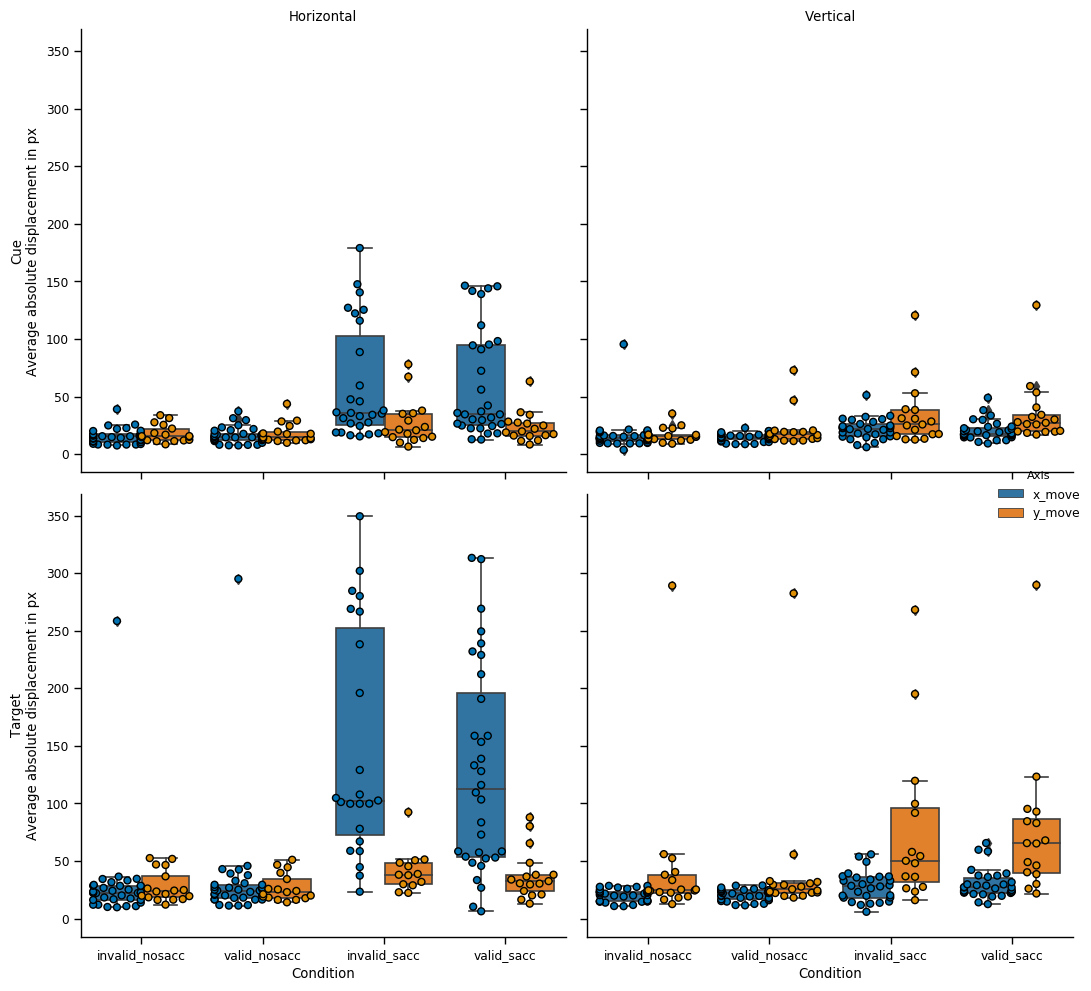


Figure 2: Visualization of the absolute deviations from the midpoint across the experiment. For each participant we calculated the average absolute eye-movement in pixels along the x – and y – axis. Data was split between valid and invalid trials and in each condition split between trials containing a saccade (“_sacc”) or not containing a saccade (“_nosacc”). For this purpose of visualization, we used the “EyeLink” saccade classification.


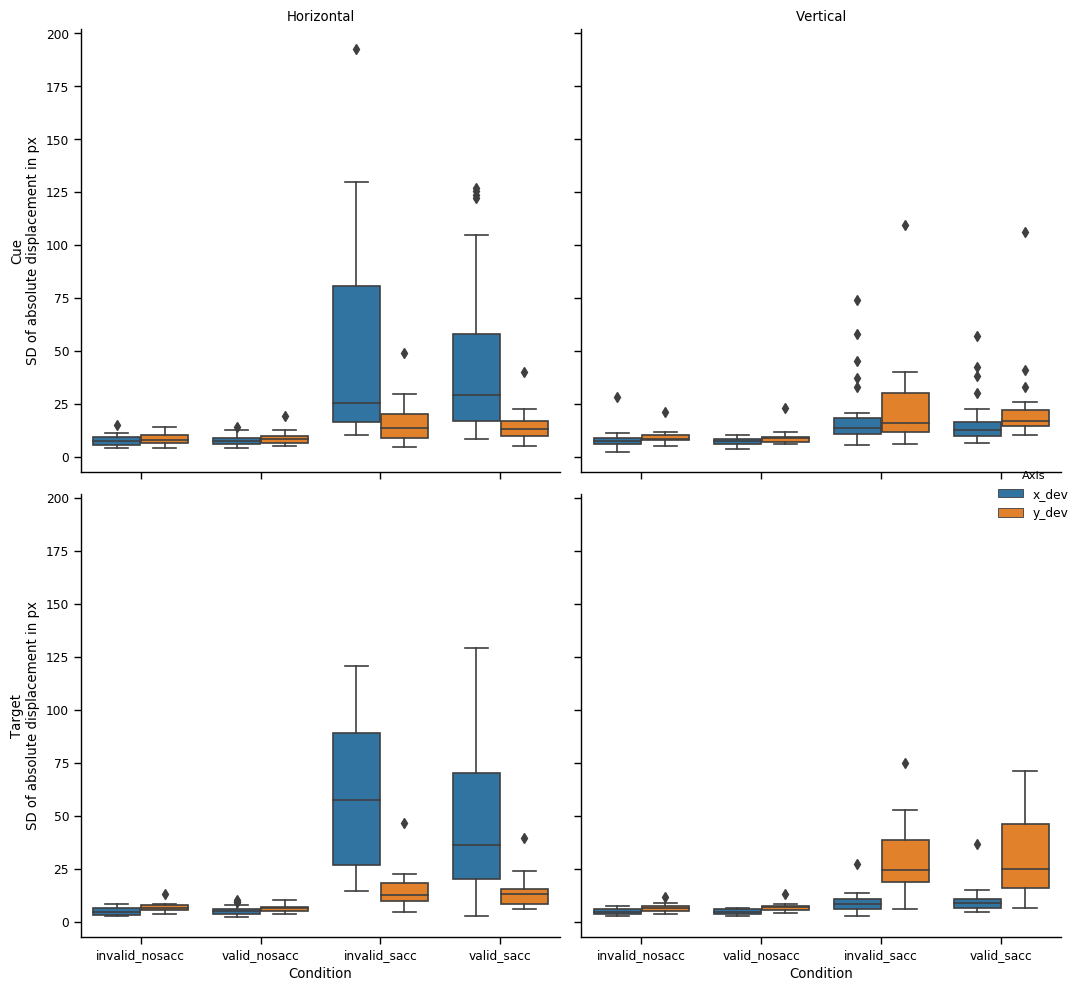


Figure 3: Visualization of the absolute deviations from the midpoint across the experiment. For each participant we calculated the standard deviation of absolute eye-movement in pixels along the x – and y – axis. Data was split between valid and invalid trials and in each condition split between trials containing a saccade (“_sacc”) or not containing a saccade (“_nosacc”). For this purpose of visualization, we used the “EyeLink” saccade classification.

# References

Abraham, Alexandre, Fabian Pedregosa, Michael Eickenberg, Philippe Gervais, Andreas Mueller, Jean Kossaifi, Alexandre Gramfort, Bertrand Thirion, and Gaël Varoquaux. 2014. “Machine Learning for Neuroimaging with Scikit-Learn.” *Frontiers in Neuroinformatics* 8.

Acland, Ben and Tom Wallis. 2016. *Cili: Cili v0.5.4*.

Avants, B., C. Epstein, M. Grossman, and J. Gee. 2008. “Symmetric Diffeomorphic Image Registration with Cross-Correlation: Evaluating Automated Labeling of Elderly and Neurodegenerative Brain.” *Medical Image Analysis* 12(1):26–41.

Cox, Robert W. 1996. “AFNI: Software for Analysis and Visualization of Functional Magnetic Resonance Neuroimages.” *Computers and Biomedical Research* 29(3):162–73.

Esteban, Oscar, Ross Blair, Christopher J. Markiewicz, Shoshana L. Berleant, Craig Moodie, Feilong Ma, Ayse Ilkay Isik, Asier Erramuzpe, James D. Kent, Mathias Goncalves, Elizabeth DuPre, Kevin R. Sitek, Daniel E. P. Gomez, Daniel J. Lurie, Zhifang Ye, Russell A. Poldrack, and Krzysztof J. Gorgolewski. 2018. “Poldracklab/Fmriprep: 1.1.3.”

Esteban, Oscar, Christopher J. Markiewicz, Ross W. Blair, Craig A. Moodie, A. Ilkay Isik, Asier Erramuzpe, James D. Kent, Mathias Goncalves, Elizabeth DuPre, Madeleine Snyder, Hiroyuki Oya, Satrajit S. Ghosh, Jessey Wright, Joke Durnez, Russell A. Poldrack, and Krzysztof J. Gorgolewski. 2019. “FMRIPrep: A Robust Preprocessing Pipeline for Functional MRI.” *Nature Methods* 16(1):111–16.

Fonov, Vs, Ac Evans, Rc McKinstry, Cr Almli, and Dl Collins. 2009. “Unbiased Nonlinear Average Age-Appropriate Brain Templates from Birth to Adulthood.” *NeuroImage* 47:S102.

Gorgolewski, Krzysztof J., Christopher D. Burns, Cindee Madison, Dav Clark, Yaroslav O. Halchenko, Michael L. Waskom, and Satrajit S. Ghosh. 2011. “Nipype: A Flexible, Lightweight and Extensible Neuroimaging Data Processing Framework in Python.” *Frontiers in Neuroinformatics* 5.

Gorgolewski, Krzysztof J., Oscar Esteban, David Gage Ellis, Michael Philipp Notter, Erik Ziegler, Hans Johnson, Carlo Hamalainen, Benjamin Yvernault, Christopher Burns, Alexandre Manhães-Savio, Dorota Jarecka, Christopher J. Markiewicz, Taylor Salo, Daniel Clark, Michael Waskom, Jason Wong, Marc Modat, Blake E. Dewey, Michael G. Clark, Michael Dayan, Fred Loney, Cindee Madison, Alexandre Gramfort, Anisha Keshavan, Shoshana Berleant, Basile Pinsard, Mathias Goncalves, Dav Clark, Ben Cipollini, Gael Varoquaux, Demian Wassermann, Ariel Rokem, Yaroslav O. Halchenko, Jessica Forbes, Brendan Moloney, Ian B. Malone, Michael Hanke, David Mordom, Colin Buchanan, Wolfgang M. Pauli, Julia M. Huntenburg, Christian Horea, Yannick Schwartz, Rosalia Tungaraza, Shariq Iqbal, Jens Kleesiek, Sharad Sikka, Caroline Frohlich, James Kent, Martin Perez-Guevara, Aimi Watanabe, David Welch, Chad Cumba, Daniel Ginsburg, Arman Eshaghi, Erik Kastman, Salma Bougacha, Ross Blair, Benjamin Acland, Ashley Gillman, Alexander Schaefer, B. Nolan Nichols, Steven Giavasis, Drew Erickson, Carlos Correa, Ali Ghayoor, René Küttner, Christian Haselgrove, Dale Zhou, R. Cameron Craddock, Daniel Haehn, Leonie Lampe, Jarrod Millman, Jeff Lai, Mandy Renfro, Siqi Liu, Jörg Stadler, Tristan Glatard, Ari E. Kahn, Xiang-Zhen Kong, William Triplett, Anne Park, Conor McDermottroe, Michael Hallquist, Russell Poldrack, L. Nathan Perkins, Maxime Noel, Stephan Gerhard, John Salvatore, Fred Mertz, William Broderick, Souheil Inati, Oliver Hinds, Matthew Brett, Joke Durnez, Arielle Tambini, Simon Rothmei, Sami Kristian Andberg, Gavin Cooper, Ana Marina, Aaron Mattfeld, Sebastian Urchs, Paul Sharp, K. Matsubara, Daniel Geisler, Brian Cheung, Andrew Floren, Thomas Nickson, Nicolas Pannetier, Alejandro Weinstein, Mathieu Dubois, Jaime Arias, Claire Tarbert, Kai Schlamp, Kesshi Jordan, Franz Liem, Victor Saase, Robbert Harms, Ranjeet Khanuja, Kornelius Podranski, Guillaume Flandin, Dimitri Papadopoulos Orfanos, Isaac Schwabacher, Daniel McNamee, Marcel Falkiewicz, John Pellman, Janosch Linkersdörfer, Jan Varada, Fernando Pérez-García, Andrew Davison, Dmitry Shachnev, and Satrajit Ghosh. 2017. “Nipype: A Flexible, Lightweight And Extensible Neuroimaging Data Processing Framework In Python. 0.13.1.”

Greve, Douglas N. and Bruce Fischl. 2009. “Accurate and Robust Brain Image Alignment Using Boundary-Based Registration.” *NeuroImage* 48(1):63–72.

Jenkinson, Mark, Peter Bannister, Michael Brady, and Stephen Smith. 2002. “Improved Optimization for the Robust and Accurate Linear Registration and Motion Correction of Brain Images.” *NeuroImage* 17(2):825–41.

Jones, Eric, Travis Oliphant, Pearu Peterson, and others. 2001. *SciPy: Open Source Scientific Tools for Python*.

Kuznetsova, Alexandra, Per B. Brockhoff, and Rune H. B. Christensen. 2017. “**LmerTest** Package: Tests in Linear Mixed Effects Models.” *Journal of Statistical Software* 82(13).

Morey, Richard D. and Jeffrey N. Rouder. 2018. *BayesFactor: Computation of Bayes Factors for Common Designs*.

Power, Jonathan D., Anish Mitra, Timothy O. Laumann, Abraham Z. Snyder, Bradley L. Schlaggar, and Steven E. Petersen. 2014. “Methods to Detect, Characterize, and Remove Motion Artifact in Resting State FMRI.” *NeuroImage* 84:320–41.

R Core Team. 2018. *R: A Language and Environment for Statistical Computing*. Vienna, Austria: R Foundation for Statistical Computing.

Treiber, Jeffrey Mark, Nathan S. White, Tyler Christian Steed, Hauke Bartsch, Dominic Holland, Nikdokht Farid, Carrie R. McDonald, Bob S. Carter, Anders Martin Dale, and Clark C. Chen. 2016. “Characterization and Correction of Geometric Distortions in 814 Diffusion Weighted Images” edited by J. Najbauer. *PLOS ONE* 11(3):e0152472.

Tustison, Nicholas J., Brian B. Avants, Philip A. Cook, Yuanjie Zheng, Alexander Egan, Paul A. Yushkevich, and James C. Gee. 2010. “N4ITK: Improved N3 Bias Correction.” *IEEE Transactions on Medical Imaging* 29(6):1310–20.

Wang, Sijia, Daniel J. Peterson, J. C. Gatenby, Wenbin Li, Thomas J. Grabowski, and Tara M. Madhyastha. 2017. “Evaluation of Field Map and Nonlinear Registration Methods for Correction of Susceptibility Artifacts in Diffusion MRI.” *Frontiers in Neuroinformatics* 11.

Zhang, Y., M. Brady, and S. Smith. 2001. “Segmentation of Brain MR Images through a Hidden Markov Random Field Model and the Expectation-Maximization Algorithm.” *IEEE Transactions on Medical Imaging* 20(1):45–57.
